# The Color of Debt: Racial Disparities in Anticipated Medical Student Debt in the United States

**DOI:** 10.1371/journal.pone.0074693

**Published:** 2013-09-03

**Authors:** Robert A. Dugger, Abdulrahman M. El-Sayed, Anjali Dogra, Catherine Messina, Richard Bronson, Sandro Galea

**Affiliations:** 1 Columbia University Mailman School of Public Health, New York, New York, United States of America; 2 Stony Brook University School of Medicine, Stony Brook, New York, United States of America; 3 Columbia University College of Physicians and Surgeons, New York, New York, United States of America; Northwestern University, United States of America

## Abstract

**Context:**

The cost of American medical education has increased substantially over the past decade. Given racial/ethnic inequalities in access to financial resources, it is plausible that increases in student debt burden resulting from these increases in cost may not be borne equally.

**Objective:**

To evaluate racial/ethnic disparities in medical student debt.

**Design, Setting, and Participants:**

Authors collected self-reported data from a non-representative sample of 2414 medical students enrolled at 111/159 accredited US medical schools between December 1^st^ 2010 and March 27^th^ 2011. After weighting for representativeness by race and class year and calculating crude anticipated debt by racial/ethnic category, authors fit multivariable regression models of debt by race/ethnicity adjusted for potential confounders.

**Main Outcome Measures:**

Anticipated educational debt upon graduation greater than $150,000.

**Results:**

62.1% of medical students anticipated debt in excess of $150,000 upon graduation. The proportion of Blacks, Whites, Hispanics, and Asians reporting anticipated educational debt in excess of $150,000 was 77.3%, 65.1%, 57.2% and 50.2%, respectively. Both Black and White medical students demonstrated a significantly higher likelihood of anticipated debt in excess of $150,000 when compared to Asians [Blacks (OR = 2.7, 1.3–5.6), Whites (OR = 1.7, 1.3–2.2)] in adjusted models.

**Conclusion:**

Black medical students had significantly higher anticipated debt than Asian students. This finding has implications for understanding differential enrollment among minority groups in US medical schools.

## Introduction

Over the past decade, the cost of medical education has increased substantially [1]–[3]. Many institutions have more than doubled their tuition fees since 1998 [1]. Between 1999 and 2010, the median educational debt and the median 4-year cost of medical school attendance grew at nearly twice the rate of inflation [4]. Since 2000, increases in medical school tuition have outpaced increases in the consumer price index, physician compensation, and overall financial aid [1]. A recent Association of American Medical Colleges (AAMC) analysis describing changes in indebtedness trends reports 2010 and 2011 mean medical student educational debt at $157,000 and $161,300 respectively [5]. While congressional efforts to address growing national undergraduate student debt have been enacted, there have been relatively few recent policy efforts addressing the growing debt burden among medical students [6].

Concomitant with this explosion in medical school tuition has been an exacerbation in racial and ethnic inequalities in matriculation into US medical schools. While minority students now constitute approximately 40% of the enrolled US medical student population [7]–[10], increases in minority enrollment have not been proportional across minority groups. In fact, since 2004, the overall percentage of Black enrollment in Allopathic and Osteopathic medical schools has fallen (2004 = 7.4% versus 2011 = 7.0%, in Allopathic institutions; 2004 = 4.0% versus 2012 = 2.7%, in Osteopathic institutions) [11], [12]. Meanwhile, enrollment of Hispanic and Asian students continues to rise [11]–[13]. Combined US continental enrollment data from the AAMC and the American Association of Colleges of Osteopathic Medicine (AACOM) for 2010–2011 reports enrollment of Whites at 58,846 (60%), Asians at (21%), Hispanic/Latinos at 6,873 (7%) and Blacks at 6,089 (6%) [7]–[10]. Compared to the overall US population, Asian students are overrepresented in the medical student population by over 75%, while Black students are underrepresented by over 100% [14].

Racial and ethnic disparities in socioeconomic status are well documented in the US, and Black families have less income and overall wealth than other racial and ethnic groups [15]–[18]. It is plausible that the growing financial burden of medical school, combined with persistent limited access to financial resources among Black families in the US, contributes to some of the decrease in the proportion of Black students enrolling in medical school. However, that hypothesis would imply that the financial burden of medical education should be higher among Black students. In that respect, we explored racial/ethnic disparities in medical student debt among a sample of over 2% of medical students in the US from among 111 of the 159 medical colleges in the US in 2010–2011.

## Methods

### Data

Our team adapted the survey instrument from a previously published New England Journal of Medicine study evaluating physician perspectives [19]. We conducted two cycles of cognitive interview pretesting with 9 and 7 students, respectively and used the feedback from these sessions to revise the items for consistency and clarity.

We then obtained written consent and self-reported data from 2414 medical students enrolled at 111 of the 159 accredited US medical schools within the 50 United States. To recruit our sample, we did the following: First, representatives to the American Medical Association (AMA), Organization of Student Representatives (OSR), Council of Osteopathic Student Government Presidents (COSGP) and faculty deans were mailed participation information (1183 total contacts). Second, representatives were asked to forward the secured confidential electronic survey and consent form to their respective medical student bodies. Third, respondents self-administered the surveys between December 1^st^ 2010 and March 27^th^ 2011. As an incentive for participation, respondents were entered into a drawing for a $100 gift card.

Respondents were excluded from the study if their responses were incomplete, they provided duplicate contact information for incentive distribution, they did not identify as a 1st, 2nd, 3rd or 4th year medical students (i.e., they were taking time off or pursuing a second degree), or did not attend a medical school in the United States. 97.6% of responses met study inclusion criteria (n = 2355).

Our primary outcome of interest was anticipated educational debt upon graduation (analyzed as a binary variable denoting anticipated educational debt above or below $150,000). Responses were collected about self-reported race/ethnicity (White, Black, Hispanic/Latino, Asian, Other). Responses indicating multiple race/ethnicity were recoded to the category “Other”. Racial/ethnic categories were based on AAMC and AACOM racial/ethnic categorization. Data were also collected about gender (male, female), age (analyzed as a categorical variable; <25 years of age, ≥25 years of age), political self-characterization (moderate, liberal, conservative/other), level of medical school (1st year, 2nd year, 3rd year, 4th year), clinical level (preclinical, clinical), category of medical school (public, private), medical school region (South, Midwest, Northeast, West). We also asked each medical student “were you or a loved one significantly affected in the recent economic downturn?” (analyzed as a categorical variable denoting presence or absence of the recession's effect). All data were electronically entered by respondents via a secure socket layer (SSL) service and stored securely.

### Analysis

First, we used AAMC [7], [9], [12] and AACOM [8], [11], [20] data about the MD and DO school enrollment totals in the 2010–2011 academic year to check our sample for representativeness and to weight accordingly by race and class year in medical school. Second, we calculated univariate statistics and used two-tailed chi-square tests to identify significant associations (α = 0.05) between each covariate of interest and race/ethnicity, as well as between each covariate of interest and the outcome (anticipated educational debt greater than or less than $150,000). Third, we fit multivariable logistic regression models of our outcome by race/ethnicity as well as each covariate associated with both race and debt at the α = 0.20 level. Fourth, a final multivariable logistic regression model of debt was fit including all covariates. All analyses accounted for weighting and were conducted using SAS (version 9.2).

### Ethics Statement

This study was reviewed and given written approval by the Stony Brook Committees on Research Involving Human Subjects. Data available upon request from the lead author.

## Results

Table 1 shows the demographic characteristics of our study sample and the estimated combined US Allopathic and Osteopathic medical student enrollment. More than 2% of the total estimated 2010–2011 US medical student population participated in the study (n = 2355/98,197). 62.1% of respondents reported anticipated educational debt in excess of $150,000 (table not shown).

**Table 1:** Combined Allopathic and Osteopathic medical student population and sample demographics, 2010–2011.

Characteristic	Sample frequency	Weighted percent	Estimated percent in the medical student population*
Female Sex	1177	51.2	47.3
Self Described Race and or Ethnicity			
White	1752	57.9	59.9
Asian	322	21.3	21.2
Black	51	7.3	6.2
Hispanic/Latino	62	8.0	7.0
Other	168	5.5	5.6
Level of Medical School			
1st year	759	25.1	26.8
2nd year	737	24.7	25.5
3rd year	428	25.0	24.4
4th year	431	25.2	23.2
Category of Medical School			
Public	1052	45.7	50.9
Private	1303	54.3	49.1
Medical School Region			
South	335	15.1	32.4
Midwest	748	29.1	24.8
Northeast	668	29.5	28.6
West	604	26.4	14.2

*Data compiled from AAMC and AACOM records of 2010–2011 total student enrollment. Percentages may not total 100 due to rounding. Self-Described Race and or Ethnicity accepted more than one answer for respondents and population MD students whereas data for DO students collapsed multiple races/ethnicities into the other category. Student population data includes levels other than 1st-4th year. Population data includes schools in Puerto Rico whereas the sample does not. MD student level population derived from the matriculating student questionnaire which also includes non-US accredited matriculation and also has augmented enrollment totals.

**Figure 1:**
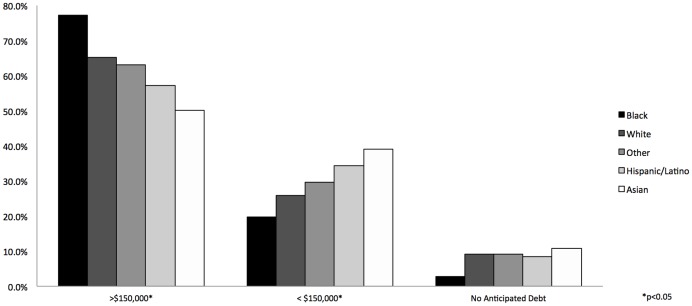
Weighted distribution of anticipated educational debt across race/ethnicity among 2355 medical students. *p<0.05.

Figure 1 shows the distribution of debt by race and ethnicity. Asian students anticipated the lowest levels of debt; 50.2% of Asian respondents reported anticipated debt greater than $150,000. Hispanic/Latinos were slightly more indebted with 57.2% reporting anticipated debt in excess of $150,000. 63.2% of students identified as Other and 65.1% of Whites anticipated debt in excess of $150,000. On average, Black students anticipated the greatest debt with 77.3% reporting debt greater than $150,000.

Table 2 shows descriptive statistics and chi-square tests between each covariate and race/ethnicity. In stratified analyses, age, gender, political orientation, self-identification as having been affected by the recession, public or private category of medical school, and school region were associated with race/ethnicity. Black, Hispanic/Latino, and Other respondents reported being affected by the recession more than those not affected (26.6% affected versus 15.2% not affected).

**Table 2:** Descriptive statistics and chi-square tests between covariates of interest and race/ethnicity among 2355 medical students.

	Race/Ethnicity					
Variables	White	Asian	Black	Hispanic/Latino	Other	?^2^ p*
Age						<0.01
<25	50.3	31.6	5.6	7.8	4.8	
>24	61.7	16.2	8.2	8.1	5.9	
Gender						0.01
Male	60.8	19.7	4.8	8.9	5.9	
Female	55.1	22.9	9.7	7.2	5.1	
Political orientation						<0.01
Moderate	54.9	23.9	9.3	6.6	5.2	
Liberal	50.9	24.5	8.3	11.3	5.0	
Conservative/Other	77.6	10.1	1.8	3.6	6.9	
Affected by the recession						<0.01
Yes	54.0	19.3	9.7	10.0	6.9	
No	61.6	23.2	5.1	6.0	4.1	
Anticipated debt						<0.01
No educational debt	59.8	25.7	2.4	7.7	4.4	
Less than $$100,000	47.2	31.1	6.6	9.6	5.4	
$100,000 - $200,000	56.3	22.7	6.0	9.2	5.8	
$200,000 - $300,000	62.3	15.4	10.3	6.2	5.9	
Greater than $300,000	65.6	16.8	6.0	8.2	3.4	
Level						0.53
1st year	55.7	24.3	7.2	6.7	6.1	
2nd year	58.7	22.4	4.9	8.6	5.5	
3rd year	57.8	21.3	6.8	8.4	5.7	
4th year	59.3	17.3	10.4	8.3	4.7	
Clinical level						0.28
Preclinical	57.2	23.3	6.0	7.6	5.8	
Clinical	58.6	19.3	8.6	8.3	5.2	
School category						<0.01
Public	55.3	22.0	5.2	11.2	6.3	
Private	60.1	20.7	9.2	5.3	4.8	
School region						<0.01
South	53.7	14.7	18.6	8.3	4.8	
Midwest	68.5	16.4	5.0	4.7	5.4	
Northeast	54.6	26.8	7.7	7.0	3.9	
West	52.3	24.4	3.2	12.5	7.7	

*χ^2^ p is the calculated chi-square value.

Table 3 shows -square tests between covariates of interest and anticipated educational debt above or below $150,000. Race/ethnicity was a significant predictor of anticipated educational debt (p<0.001). There were also significant associations between age, self-identification as affected by the recession, and the public or private status of schools, with debt. However, level in school, region, and gender were not associated with the outcome.

**Table 3:** Descriptive statistics and chi-square tests between covariates of interest and anticipated educational debt among 2355 medical students.

	Anticipated educational debt		
Variables	<$150 k	>$150 k	?^2^ p*
Age			<0.01
<25	45.7	54.4	
>24	34.1	65.9	
Gender			0.711
Male	38.4	61.6	
Female	37.5	62.5	
Race			<0.01
White	34.9	65.1	
Asian	49.8	50.2	
Black	22.7	77.3	
Hispanic/Latino	42.8	57.2	
Multiple/Other	36.8	63.2	
Political orientation			0.13
Moderate	39.4	60.6	
Liberal	38.9	61.1	
Conservative/Other	33.4	66.6	
Affected by the recession			<0.01
Yes	30.9	69.1	
No	44.7	55.3	
Level			0.85
1st year	36.8	63.2	
2nd year	37.1	62.9	
3rd year	38.5	61.5	
4th year	39.4	60.6	
Clinical level			0.39
Preclinical	36.9	63.1	
Clinical	38.9	61.1	
School category			<0.01
Public	45.7	54.3	
Private	31.4	68.6	
School region			0.53
South	37.8	62.2	
Midwest	37.8	62.2	
Northeast	40.3	59.7	
West	35.5	64.5	

*χ^2^ p is the calculated chi-square value.

Table 4 shows both crude and adjusted logistic regression models of debt above $150,000 by race/ethnicity. In the crude model, Black students, (OR 3.4, 95% confidence interval (CI): 1.6–6.7), White students (OR: 1.9 [95% confidence interval (CI): 1.4–2.4]) and students in the Other ethnic category (OR: 1.7 [95% confidence interval (CI): 1.1–2.6]) reported significantly higher likelihoods of anticipated debt greater than $150,000 relative to Asians. In the trivariate logistic regression models of debt above $150,000 by race/ethnicity (not shown), none of the adjusted covariates attenuated the association between race/ethnicity and debt to non-significance among any of the racial or ethnic groups. In all the models, Hispanic students did not have a significantly different likelihood of anticipated debt greater than $150,000 when compared to Asians. In the fully adjusted model, there remained a significant association between race/ethnicity and debt among Blacks and Whites relative to Asians. Black respondents had the highest likelihood of debt greater than $150,000 in the fully adjusted model when compared to Asians (OR = 2.7, 1.3–5.6). White respondents had the next highest likelihood of debt significantly greater than $150,000 in the fully adjusted model when compared to Asians (OR = 1.66, 1.28–2.16). There was no significant difference in likelihood of debt greater than $150,000 between Black and White students in the fully adjusted model.

**Table 4:** Multivariable logistic regression models of anticipated medical student debt greater than $150,000 by race/ethnicity among 2355 medical students.

	Unadjusted		Adjusted	
	OR	95% CI	OR	95% CI
Race				
Asian (Reference)				
Hispanic/Latino	1.3	0.8–2.4	1.3	0.7–2.3
Other	1.7	1.1–2.6	1.5	1.0–2.2
White	1.9	1.4–2.4	1.7	1.3–2.2
Black	3.4	1.6–6.7	2.7	1.3–5.6
School Category				
Public (Reference)				
Private			1.8	1.5–2.2
Political Orientation				
Moderate			0.8	0.6–1.1
Liberal			0.8	0.6–1.0
Conservative/Other (Reference)				
Recession Hit				
Yes (Reference)				
No			0.6	0.5–0.7
Age				
<25			0.7	0.6–0.9
≥25 (Reference)				

## Discussion

In a sample of over 2% of the US medical student population we found that 62.1% of medical students anticipated educational debt in excess of $150,000 upon graduation. We found that the proportion of Blacks, Whites, Hispanics and Asians reporting anticipated educational debt in excess of $150,000 was 77.3%, 65.1%, 57.2% and 50.2% respectively. After adjusting for potential confounders, both Black and White medical students demonstrated a significantly higher likelihood of anticipating debt in excess of $150,000 when compared to Asians [Blacks (OR = 2.7, 1.3–5.6), Whites (OR = 1.7, 1.3–2.2)], although Hispanics did not differ in likelihood of anticipated debt in excess of $150,000 compared to Asians, and Blacks did not differ in likelihood compared to Whites.

Our findings extend the extant literature in important ways, contributing unique insight into disparities in medical student debt. First, we included data about medical students from both Allopathic and Osteopathic institutions in order to better reflect the total population of students entering the physician workforce, uncommon in studies about medical student debt. Second, we were able to study Black and Hispanic students separately, uncovering substantial heterogeneity in their debt. Third, we sampled 1^st^ through 4^th^ year medical students whereas previous studies have almost exclusively evaluated debt levels among students at the point of graduation. Fourth, to the authors, this is the first known work utilizing multivariable analysis that demonstrates the significant relationship between medical student race and level of indebtedness.

Although we are not aware of combined Allopathic and Osteopathic studies at a scale comparable to this work, our demonstration of relative debt burden across racial/ethnic group is consistent with previous work, conducted in 2010 in three Allopathic medical schools that found that Asian students anticipated a significantly lower average debt burden at graduation than Caucasians and under-represented minorities (URMs; [Black and Hispanic students]) ($113,000 versus $138,000 and $162,000 respectively) [21]. A study by Jolly and colleagues that analyzed data collected by the AAMC's 2003 survey of Allopathic graduating medical students, demonstrated that Asians, Puerto Ricans, and Mexicans in 2003 graduated with an equivalent average debt burden of $100,000 whereas Black medical students had the highest debt burden followed closely by Whites [22]. Similarly, the AACOM 2011 survey of Osteopathic graduating seniors found that Asians had the lowest mean educational debt ($187,979) while Blacks had the highest mean debt ($217,659) [23]. Although there was greater parity in the mean debt of White ($210,267) and Hispanic ($211,659) students in Osteopathic medical schools, the report also demonstrated that 100% of Blacks surveyed reported having educational debt [23].

Our findings regarding racial and ethnic disparities in anticipated debt among medical students reflect well our understanding of racial/ethnic differences in socioeconomic status in the US. Studies have demonstrated that in general, students from relatively low-income families have a greater likelihood of incurring high educational debt [21], [24]. In this regard, we found that disparities in medical student debt burden roughly correlated with racial and ethnic disparities in income [25], suggesting that the relationship between race/ethnicity and access to financial resources may be the central driver of the observed relations. Importantly, however, we found that Hispanics were a notable exception to this general relationship, as Hispanics in the US have among the lowest median incomes, yet Hispanic medical students experienced comparatively low anticipated educational debt [25]. Along with Hispanics, Asian medical students had low anticipated educational debt. Hispanic medical students, like their Asian counterparts, are more likely to come from immigrant households. It is plausible that immigrant families may be less comfortable with the American norm of educational loan utilization than non-immigrant families [26]. Moreover, as is suggested among Asians [25], [27], parental education may be an important underlying determinant of child educational debt among immigrant families; educated parents are likely to be 1) wealthier and therefore better able to finance their children's education, and 2) more likely to recognize the value of education. In this way, they may be more willing to offset the costs of their children's graduate education.

Our findings may have important implications with respect to the changing demographics of US medical students. We found that Black students had higher debt burdens than their counterparts from other racial and ethnic backgrounds. It is plausible that this disproportionate debt burden may play a role in the decline in medical school attendance among Black students.

The literature has established that high medical student debt has frustrated efforts to create a diverse and representative physician workforce [1], [3], [28]–[30]. The cost of medical education has also been shown to deter qualified Black and Hispanic applicants, in particular [22], [31]. For example, a recent national survey found that cost was a major deterrent for qualified potential applicants, and it was the single most important deterrent among Blacks and Hispanics [22]. However, while the high cost of education may deter both groups, higher anticipated debt among Black compared to Hispanic students may explain, in part, why matriculation among Blacks is decreasing in the setting of increasing matriculation among Hispanics [11]–[13], [20]. However, the racial difference in debt burden is only one potential mechanism, of potentially many, such as differences in application and acceptance rates, which may explain racial disparities in medical school matriculation.

There are several limitations that should be kept in mind when interpreting our findings. First, our study evaluated anticipated graduating debt burden in students prior to their graduation, rather than actual debt after graduation. As a result, our outcome of anticipated debt may not accurately represent student debt burdens at the time of graduation. Also, respondents may not accurately report levels of anticipated debt. Nonetheless, the growth in anticipated educational debt our findings report is consistent with the debt reported by recent graduates [32]. Second, our survey was not nationally representative. In that respect, our findings may be subject to considerable selection bias, which may have influenced our findings. For example, it is possible that students with the most debt may have been more likely to participate than counterparts with less debt. However, as student debt was not an explicit focus of the student survey, students would have not have had particular reason to participate differentially based on debt level. Moreover, our sample is not generalizable to the overall US population of medical students. However, we weighted our sample by race and class year to improve the representativeness of the findings. Furthermore, our findings corroborate and build upon evidence in other studies showing similar distributions of debt by race and ethnicity.

## Conclusions

Our work has important implications for health policy and further research. First, our work suggests that the burden of medical student debt is substantial, and that the distribution of debt across race and ethnicity is disproportionate. Importantly, this disproportionate burden may be influencing the diversity of the physician workforce [21], [22]. As concerns over the supply of physicians grow, further research evaluating the influence of medical education cost on the physician supply is needed [33]. Second, while our work demonstrated that Blacks bear the highest burden of medical student debt, research is needed to assess the sequelae of this inequality in medical student debt. For example, as discussed, higher debt burden may be driving the current downward trend in Black medical student matriculation, posing questions ripe for future consideration. Third, future research is also needed to identify the etiology of racial and ethnic disparities in medical student debt. For example, future work is needed to understand how disproportionalities in family income, immigration status, financial aid, and merit scholarship aid may influence disparities in medical student debt. Fourth, investigations into the relatively high matriculation and low debt of Hispanics in comparison to other minority groups may be fruitful.
